# “Helpful help” in vocational support for emerging adults not in education, employment, or training. Staff and participant perspectives

**DOI:** 10.3389/fpsyt.2025.1511255

**Published:** 2025-05-16

**Authors:** Kristin Berre Ørjasæter

**Affiliations:** Faculty of Nursing and Health Sciences, Nord University, Namsos, Norway

**Keywords:** art-based vocational rehabilitation, healing environment, NEET, personalized follow up, reflective lifeworld research, qualitative study

## Abstract

**Introduction:**

Aiding emergent adults not in education, employment, or training (NEET) presents a profound challenge.

**Methods:**

This article, based on reflective lifeworld research, explores the defining elements of helpful help within two art-based vocational rehabilitation programs in the context of the Norway’s Labor and Welfare Service (NAV). Interviews were conducted with 11 emerging adults with social and mental health issues and three NAV staff members skilled in art-based vocational rehabilitation.

**Results:**

“Helpful help” is a dynamic, multifaceted phenomenon involving the three defining elements: balancing demands and support for personal growth, a healing environment with supportive staff, and tailored facilities.

**Discussion:**

These three elements intertwine within the individual’s lifeworld, emphasizing that help unfold through their personal progression, shaped by staff attunement and a nurturing atmosphere. This phenomenon flourishes in environments of optimism and nurturance, where staff engage authentically, fostering supportive and non-judgmental relationships. By embracing a strength-based approach, these spaces become welcoming and enhance well-being. Emergent adults with social and mental health issues need ongoing and personalized support that emphasizes peer interactions and focuses on building self-belief and social skills, beyond job opportunities.

## 1 Introduction

The transition from adolescence to adulthood, often called “emergent adulthood,” constitutes a critical stage of development in young people’s lives (1). Emergent adulthood spans ages 18 to 29 and is distinctive from both adolescence and adulthood (2). During this phase, individuals experience relative freedom from social roles and expectations, enabling them to explore diverse paths in relationships, careers and personal belief. It is characterized by “a high degree of exploration and uncertainty about future, offering more opportunities for independent exploration than any other life stage” (3). For some, emergent adulthood proves to be particularly challenging and may lead to dropout from school or from employment (4).

One significant challenge during this period is the risk of becoming a NEET (Not in employment, Education, or Training). This term refers to young people who are neither working nor studying (5), highlighting the difficulties some face in navigating this transitional stage of development. Risk factors for becoming NEET include low socioeconomic status, school dropout, poor mental health, psychosocial problems, and being a former recipient of child welfare services (6). The challenges faced by NEET individuals often lead to (7) feelings of shame, rejection, and dependence on public benefits (8), as well as increased risk of unfavorable life trajectories (9), poorer mental health, and substance use (10).

Norway has a lower proportion of NEET individuals compared to other European countries. However, these individuals tend to receive more health-related benefits, have poorer mental health, and are less educated than their European peers (11). Research indicates that labor market interventions for young unemployed individuals often fails to achieve positive results (12, 13), despite good intentions (14–16). The needs of emerging adults with NEET status have frequently been underestimated, leading to suboptimal outcomes from previous investments (32). Consequently, these individuals harbor low expectations about the effectiveness of assistance from the Norwegian Labour and Welfare Administration (NAV) (13), which has been criticized for its passive follow-up approach, prolonged response times (17, 18), and poorly adapted services (12). The challenges NEETs face in obtaining optimal treatment and support are not unique to Norway. Recent UK-based research indicates that NEETs tend to have poorer treatment outcomes compared to their peers, although increased session attendance improves their recovery prospects (19).

Addressing these challenges requires a nuanced understanding of what constitutes “helpful help” for emergent adults with NEETs status experiencing social and mental health issues. While helping involves assisting someone out of a difficult situation, it does not always align with the individual’s desires or needs (20). The concept of “helpful help” encompasses various forms of assistance, support, and intervention perceived as significant or beneficial by individuals. Generally, helpful help includes all actions and outcomes when people assist others. Although the perception of helpfulness varies depending on one’s perspective and beliefs about the purpose of help, the concept of helpful help typically challenges prevailing assumptions by asserting that assistance from services does not yield beneficial outcomes (21).

“Helpful help” is often associated with philosopher Søren Kierkegaard and his idea of the “secret of the art of helping”, which emphasizes the importance of seeing the individual where they are as a starting point for helpful assistance. To be helpful, the helper must simultaneously understand the individual’s circumstances and possess additional insight (20). Self-determination theory (22) provides a foundation for understanding the dynamics of help and its effects on individuals. Bamberger et al. (23) highlight that help must empower recipients, enhancing their competence and autonomy. This is also emphasized by Lee et al. (24), who categorize help into empowering and nonempowering types. Empowering help is characterized by the recipient being an active participant in the assistance process, while nonempowering help involves the recipient being more passive. In the former, help is seen as a mastery experience, where the helper invests both energy and effort, indicating that the recipient is valuable and capable. However, helpful help can be understood as a complex concept, where its positional nature must be considered. This involves analyzing who perceives the help as helpful – the helper or the recipient (25).

When it comes to mental health, perspectives on what constitute helpful help are divided. Professionals often focus on treating individual causes, frequently overlooking economic factors, employment, and relationships. In contrast, recipient and their families emphasize the importance of personal and practical support, often favoring informal aid over professional services (21, 26–28). Holland et al. (27) found that young people who self-harm consider informal support from friends, pets and family as most helpful, alongside self-help techniques and strategies. Conversely, social services were seen as the least helpful by these youngsters. Other studies indicate that the relationship between helper and recipient is crucial for the perceived helpfulness of support. Sommer et al. (29) report that supportive interpersonal relationships are essential for young people’s participation in education and employment. Natland et al. (20) highlights that such relationships help create the cognitive surplus needed for success in labor and welfare services. Katznelson (30) emphasizes that motivation for education among young people on the margins of the labor market and educational system should be viewed as a process influenced by their interaction with their environment, rather than an individual trait.

Given limited knowledge about what constitutes helpful help, it is essential to gather insights from both emerging adults and staff within labor and welfare services who have engaged with individuals facing social and mental health issues. To that end, the study presented in this article aimed to explore the characteristics of helpful help within labor and welfare services by answering the following research question: What are the defining elements of helpful help as experienced by NEET individuals and staff in art-based vocational rehabilitation programs within labor and welfare services?

## 2 Materials and methods

### 2.1 Reflective lifeworld research

The study followed the reflective lifeworld research (RLR) approach developed by Dahlberg et al. (31), which generally seeks to describe the phenomenon being studied from the perspective of the research’s participants. By focusing on participants’ lived experiences, RLR provides a useful lens for exploring the depth, context, and nuances of phenomena such as helpful help. By embracing subjectivity and recognizing the lifeworld of human beings, RLR enriches understandings of such complex phenomena. The core concept of RLR, the lifeworld, is considered the fundamental context of human experience. It encompasses the everyday, pre-reflective experiences and meanings that individuals live through (31). This is the world experienced in the subjectivity of everyday life: the world that people live in, that they take for granted on a daily basis, and that they are familiar with and do not question (32). Therefore, RLR has the potential to deepen understandings of phenomena by embracing subjectivity and acknowledging the lifeworld of human beings (31).

### 2.2 Art-based vocational rehabilitation programs targeting youth

The study was conducted within vocational rehabilitation programs at two job centers targeting youth, overseen by labor and welfare services. Escorpizo et al. (33) propose a global definition of vocational rehabilitation as “a multi-professional evidence-based approach that is provided in different settings, services, and activities to working age individuals with health-related impairments, limitations, or restrictions with work functioning, and whose primary aim is to optimize work participation”. Given that the arts are the core activities designed to “assist young people in overcoming barriers to education and employment” (34), this study refers to these vocational rehabilitation programs as art-based vocational rehabilitation (ABVR).

A provisional definition of ABVR is a specialized approach to vocational rehabilitation that utilizes tailored art-based activities to enhance individuals’ competencies, work readiness, and self-belief, thereby increasing opportunities for education and employment while reducing barriers to these goals.

In light of the global concern regarding youth mental health and wellbeing, there is a pressing need for innovative solutions that are accessible, scalable and cost-effective. Golden et al. (35) advocate for an increased emphasis on the potential of art-based strategies to enhance the health and wellbeing of young people. Although research on art-based programs for youth is limited, Calero et al. (36) present promising findings related to implementing of such activities in youth vocational training.

The ABVR programs in this study target individuals aged 16–30 who have withdrawn from education or employment due to social and health-related issues. The primary goals were to identify the interests and skills of these individuals and facilitate their smooth transition into work, further education, or training. Staff with backgrounds as case managers and/or as musicians guided the initiatives. The art-based programs varied depending on the emergent adult and the job center, but generally included (1) music production, where participants created music, wrote lyrics, recorded tracks, and performed as part of a house band; and (2) event promotion, where they promoted events and concerts in local venues and other locations in the city and surrounding areas. Additionally, participants engaged in creative productions across the education, health, and cultural sectors at local, regional, and/or national levels. The programs, operating from 9:00 to 15:00 on weekdays, offered the flexibility to craft personalized plans that address unique issues and aided integration into work environments or educational pathways. Initially intended as short-term interventions lasting 3–6 months, the programs adapted to provide extended support for up to 1–3 years, depending on the individual’s needs and the complexity of their situation, as was the case with most NEET individuals in the study.

### 2.3 Participants

The study’s inclusion criteria targeted two groups (1): emerging adults with disabilities who have experienced ABVR and (2) staff members who have worked with emerging adults within ABVR. A purposive sample of 11 emerging adults and three staff members was formed for the study.

Participant recruitment was facilitated by two gatekeepers at NAV, who disseminated information about the study via phone to potential participants and via email to former and current participants in ABVR. The gatekeepers assessed the interest of contacted individuals in participating in the study, and if interested, then a meeting with the potential participant was arranged with the researcher. Of the emerging adults approached, 11 agreed to be interviewed, two declined, and three did not respond to the invitations.

Among the 11 young participants, 6 were men and 5 were women, with an average age of 24,9 years. Two had started a bachelor’s degree, one had completed high school, while the rest had struggled to finish. Some had attempted to start several times without success, while others had dropped out after one or two partially or fully completed years in high school. Participants reported challenges such as ADHD, PTSD, anxiety, depression, substance abuse, instability, suicidal tendencies, gaming issues, substance abuse, absenteeism, and social exclusion, and somatic complaints. Most had comorbid disorders. **Table 1** provides an overview of the participants who agreed to participate.

**Table 1:** Overview of emerging adults who participated in the study.

Background Information of the Emerging Adults (*n* = 11)				
Gender	Man	Women		
	6	5		
Age in years	20–25	26–31		
	6	5		
Experience with ABVR	0–6 months	6–12 months	1–2 years	>2 years
	1	2	5	3
Follow up from others	Mental health services	Child welfare services	No follow-up, except from NAV and general practitioner	
	3	1	7	
Main area of disability	Mental health	Addiction	Social	
	7	2	2	

In addition to the participating NEET individuals, the researcher contacted staff members, per agreement with the gatekeepers, to explore their interest in participating in the study. All contacted staff members consented to participate. At the time of data generation, all staff members had been working in ABVR for 3–8 years. While one had extensive work experience in NAV, the others had primarily gained their expertise through external vocational rehabilitation companies appointed by NAV, which had limited their direct involvement with NAV’s internal operations. All staff members were men in their 40s and 50s.

### 2.4 Data generation

For a deeper understanding of the defining elements of helpful help for emerging adults, interviews were conducted as a method of generating data. In general, interviews are suitable for generating both in-depth knowledge and a comprehensive understanding of a phenomenon about which knowledge is limited (37). Following the principles of an open RLR approach, the interviews were aimed at understanding forms of meaning in everyday life among people with lived experience with the phenomenon under investigation. For an open, reflective dialogue, the interviews were conducted with a focus on striking a balance between the structured and unstructured (31). Directly accessing the participants’ experiences with and descriptions of help in NAV involved asking questions such as “How would you describe NAV’s vocational rehabilitation program?”; “What does it mean to participate in art-based vocational rehabilitation?”; “What kind of experiences have you had with art-based vocational rehabilitation?”; “What significance does vocational rehabilitation have for you and your life?;” and “What needs to be present for art-based vocational rehabilitation to be helpful for you?” Follow-up questions such as “Would you please give an example?” and “Would you please elaborate on that?” were asked to gain an even deeper understanding of the phenomenon.

All interviews were conducted from August 2021 to August 2023, either virtually or in person at a location of the ABVR program or at the university where the researcher is employed. The interviews lasted 30–75 minutes, were audio-recorded, and were transcribed verbatim. Approximately 175 pages (i.e., Times New Roman, size 12 font, single spacing) of transcribed material was produced for the study.

### 2.5 Data analysis

A bridled attitude was adopted for data analysis, meaning the researcher consistently reflected on own understanding of the phenomenon without taking for granted what was understood, remaining present during analysis while waiting for the phenomenon to present itself and exploring possible understandings. According to Lindseth (38), because a phenomenon speaks quietly, researchers must decelerate the process of understanding and adopt more than an everyday attitude to let the phenomenon reveal itself.

In the first step, the researcher carefully read the transcripts multiple times to form an understanding of them as a whole. Reflections and questions were noted to remain open to the data and prevent confirmation bias. The RLR analysis stresses the importance of patience to allow the phenomenon to reveal its complexity (31).

In the second step, preliminary meaning units corresponding to the study’s aim were extracted from the text. These units were then related to each other, forming larger patterns of meaning, or clusters, while staying close to the original statements.

In the third step, meanings emerged thoughtfully, avoiding haste and carelessness (31). The bridling attitude controlled initial thoughts and biases, striving to encounter “otherness” and see things from new perspectives. To deepen understanding and maintain openness for the phenomenon to show itself, the researcher asked several questions throughout the analysis to increase its credibility, including “What are they expressing?”; “Why do they express it like that?”; “What does that mean?”; and “Why do I understand the material as I do?”

In the fourth step, the researcher labeled the constituents (1): Provision of sufficient time and space (2), appropriate competence and personal suitability (3), premises tailored to activities and target groups, and (4) nurturing atmosphere for growth. This labeling aimed to capture the essential meaning of the data, with both the component parts and their understanding evolving during analysis.

In the final step, the researcher drafted the article and presented it at an academic seminar with her affiliated Mental Health research group. Writing was integral throughout the analysis (39), and this final step facilitated a new understanding of the phenomenon’s essential meaning and components, informed by seminar discussions. Based on this process, the constituents were re-labelled to stress-reduced support (2), professional co-walkers (3), a healing environment, and (4) tailored facilities. After further academic reviews in this journal, making the constituents distinctive, the constituents were refined to three (1): balancing demands and support for personal growth (2), a healing environment with supportive staff, and (3) tailored facilities.

### 2.6 Ethics statement

The study received approval from the Centre for Research Data to handle personal data, and a pre-assessment request was submitted to the Committee for Medical and Health Research. However, as the research was not classified as a medical or health-related project, it was not subject to the Health Research Act and further ethical approval. All participants provided informed consent to participate. Data were treated confidentially and anonymized in accordance with social sciences and humanities guidelines (40), as well as general data protection regulations.

## 3 Results

This section delves into the essence of helpful help following the reflective lifeworld approach outlined by Dahlberg et al. (31). The essence captures the fundamental, all-encompassing nature of the phenomenon. Subsequently, the constituents are delineated such that each illuminates the various aspects of the phenomenon. Together, the essence and its constituents create a rich, detailed description of the defining elements of helpful help.

At heart, the essential meaning of the phenomenon of helpful help is acknowledged as support tailored to each emergent adult’s rhythm and necessities. Within the lifeworlds of emerging adults and staff members, the assistance provided by staff members of ABVR programs gains value when balancing demands and support for personal growth. This orientation calls for the presence of “we”-focused staff who are the personification of safety, care, and optimism and who are invested in understanding and supporting the journeys of emergent adults as they progress toward work, education, or training and, ultimately, a life imbued with meaning. These individuals require the freedom to advance at their own pace—a pace that allows them to “hurry slowly” without the constraints of rigid time and recovery-targeted requirements. Additionally, facilities must align with the activities provided and that cater to each emergent adult’s specific needs for proximity and space within a safe environment.

In what follows, the essence of helpful help in labor and welfare services is further elaborated into three constituents (1): balancing demands and support for personal growth (2), a healing environment with supportive staff, and (3) tailored facilities.

### 3.1 Balancing demands and support for personal growth

This constituent underscores the need for staff to balance demands and support, ensuring that emerging adults with social and mental health related issues, who have been excluded from work, education, and/or training, receive help that effectively addresses their unique challenges. The notion of rigid production-related demands was perceived as being overly demanding and a source of stress. For instance, Milla found comfort in the reality that demands were present but not as expectations of what she should produce. This distinction highlights the nuanced understanding of demands within the context of the individual journeys of NEET individuals:

The first requirement was to show up and be there. You didn’t have to do anything special in the program, but you had to show up. I found that there was a good balance with requirements. It wasn’t like you have to be here every day, from 9 to 3, and produce this. It wasn’t like that. It wasn’t that stressful. I wouldn’t have been able to do that either. But it was a very feasible requirement; Coming and just being. (Milla)

Participants highlighted the delicate balance between receiving clear demands and having personal space. They stressed the need for ample room in order to achieve individual growth and progress. If staff imposed too many demands or overly high ones, then emerging adults risked feeling suffocated. Conversely, if staff were too distant and became passive, then the emergent adults felt insecure and undervalued. Thus, striking the right balance between encouragement and restraint is crucial, as Milla also explained:

Had someone told me on my first day here that I had three months to manage life, I would’ve left immediately. I wouldn’t have been able to recover in three months. There has to be enough room. It’s important that they [staff members] are present and supportive but not overly pushy. You can’t stress people or force them to recover. Of course, a little push is necessary, but one has to be an extremely understanding human being to know when to push. If you push too hard, then you’ll push them away.

Participants recounted experiences where previous NAV staff had set time-bound expectations for the support that they received or the milestones that they should achieve. However, those expectations had often proven damaging to their coping experiences. Learning from those experiences, some participants chose to avoid setting goals for what they should master within a fixed time frame. Bea, for instance, expressed a desire to secure and maintain employment once she felt well. However, determining in advance when doing so would be possible added stress and risked making her feel like a failure despite her progress:

I had previously set clear goals to meet within a specific time frame. It put tremendous pressure on me; I felt that I had to recover or be able to manage certain tasks by a certain date. If I didn’t succeed, I was left with a feeling of incapacity. Now, I prefer to say that it takes as long as it takes. I have health issues and many struggles. Whether I master those things this year, next month, or in 10 years doesn’t matter. I need to take the time I need to recover. I may never succeed, but I have to try, and I know that stress won’t help me. (Bea)

The first constituent, balancing demands and support for personal growth, thus illuminates the lived experiences of navigating demands with subtlety, cultivating personal growth and progress, and balancing the interplay of support and personal space.

### 3.2 A healing environment with supportive staff

The second constituent, a healing environment with supportive staff, entails a nurturing and empowering atmosphere. This includes the presence of staff who demonstrate genuine interest, maintain a positive attitude towards those they assist, and commit to acquiring the necessary expertise to support them effectively. In this environment, staff and emergent adults collaborate as a cohesive “we”, with personal growth and individual strengthening at the forefront.

Having staff members with both the necessary music competencies and the ability to work with emergent adults facing significant adversity is crucial. While a foundational knowledge of music among staff is considered central, guidance from celebrated musicians is not a requirement. Instead, participants value staff who are humble, eager to learn, and genuinely interested in both music and the individuals themselves:

Leading a group of struggling participants doesn’t require you to be a rock star, but it does require some experience. … Sometimes, individuals with a strong passion for music can perform as well as professional artists. However, the person has to be interested in both teaching others and learning from them. (Kaira)

Participants underlined that personal qualities and relational skills were paramount. Formal education was secondary to the staff’s genuine interest and willingness to learn how to assist struggling emergent adults: “He became a god to many of us. It was peculiar. His formal education was limited, but he was an avid reader and deeply interested, which led to an increase in his competence”. (Milla)

Experiencing staff prioritizing the individuals above all else was of importance. As Kaira put it, “He was consistently there for us: plain and simple. In his priorities, we were always at the forefront, never himself. His entire focus was on us, on aiding us.”

Working with emergent adults was not seen as a role suited to everyone. Participants provided concrete evidence highlighting the critical importance of staff members’ personal suitability and the potential negative consequences of employing individuals lacking these essential qualities and skills. A notable example occurred when staff member Nigel departed, and a culturally experienced individual took over the team, leading to adverse outcomes for the participants. Kaira, a long-standing member of ABVR, shared her insights on selecting suitable staff for such programs:

The program needs to be run by someone who knows what they’re doing and treats the participants well. We noticed the difference when others came in. … I preferred working with Nigel [a staff member] because he viewed me as an individual. He created a program that suited me, that helped me. He was always good to have by our side. When he left the program and Lisa [another staff member] came, it became complete chaos. She tried to be a friend but not in a good way. We realized that she spoke unkindly about us, questioned whether we were ill, and treated us as if we were just lazy. (Kaira)

The presence of optimistic staff who acted as co-walkers for emergent adults was deemed crucial. Most participants had lost hope and confidence in their ability to access or manage work and education upon joining ABVR. Liam, among others, relied on the staff to sustain his hope.

I came in with a huge addiction problem. He [Nigel] said, “We’ll fix that. You have to do the work, and you’re going to have a hard time, but we’ll fix it.” He had that kind of faith. I didn’t, but he did. I went to a party shortly after I started the program. Someone I used to buy drugs from was there. I was quite drunk and proud that I had managed to say no to drugs. I called Nigel and exclaimed, “We’ll make it! We’ll make it!” And he said, “What is it that we’ll do?” I told him that I had said no to drugs, and he replied, “Damn straight. We’ll make it—I told you so!” Then we agreed that it might be a good idea for me to go home, to avoid failing that evening.

Participants illuminated the significance of working in an environment that was supportive, that they had become part of a collective where they could support, encourage, and care for each other. Most participants described the ABVR as their first encounter with such a work environment. Celine shared her experience: “The way we interacted with each other and fostered a positive atmosphere was by not being strict but understanding toward each other. I was unaware that such a conducive work environment existed until I came here”.

The staff were depicted as being safe, caring figures who nudged the emergent adults to take on challenges that they previously thought were beyond their capabilities: “When he [Julian, a staff member] believed that we were ready to perform on stage, we did. He didn’t force us but just presented us with a real challenge”. (Bea). The staff indeed desired to challenge all participants after recognizing that growth could emerge from those challenges. Even though playing music together was the chief activity in the programs, the primary goal was not to cultivate musicians, but to build relationships and foster in their potential. Participants showed varying level of proficiency, with some being so skilled they weren’t allowed to play their primary instrument. Julian, a staff member, explained:

We have young people who come from the conservatory of music, and of course, they don’t get to play their main instrument. They have to play a different instrument and share their knowledge. The team feeling and doing something together is what’s important here (.) Strengthening individuals is central; job assignments are given secondary priority.

The participants perceived a contrast with the typical workings of the music industry and recognized that accumulating mastery experiences takes precedence over performing concerts. David shared his experience:

We were supposed to have a concert on Friday, but things in the group fell apart. The staff member informed us that he’d canceled it because we weren’t ready. One of us couldn’t handle the pressure of being the only vocalist. He realized it might not go well and canceled to avoid risking the vocalist’s well-being. That prioritization of people over performance is a stark contrast to the “show must go on” mentality of the music industry.

Having participants experience success was indeed prioritized. The staff described their overarching role in ensuring that emergent adults experience mastery and avoid situations with a low likelihood of success and a high risk of failure. Julian reflected on that focus: “Participants aren’t allowed to perform if there’s a chance that they might fail. They need to succeed.”

Staff members Julian and Nigel emphasized the importance of maintaining continuity within the group to preserve the desired environment. Julian noted: “I can’t completely replace all participants because it’s important to have some continuity bearers who know how it should be, who can pass that mentality on.” Nigel, concurred, recalling negative consequences from a past instance where nearly all participants were replaced simultaneously. He stressed the value of retaining a core group familiar with the program:

Ninety-five percent of the group departed simultaneously, which had us starting nearly from scratch. Then, there was a void. We felt it very keenly. For the next 3–4 months, we were utterly inactive. Nothing happened. It was an absolute crisis. (Nigel)

This constituent emphasizes the importance of a thriving work environment with competent staff who nurture interest, well-being and growth of emergent adults, prioritize providing genuine help and contrast with the norms of the music industry.

### 3.3 Tailored facilities

The third and final constituent, tailored facilities, highlights the need for customized resources suited to specific activities and the target group. Participants underscored how the location and design of these facilities outside the typical NAV’s office landscape were much more preferable. Staff and emergent adults particularly noticed that this changed the dynamics of giving and receiving help.

In both ABVR programs, NAV rents the facilities during the day, while they remain open for other purposes in the afternoons, evenings, and weekends (e.g., town halls, concert halls, and leisure centers for young people). Staff members observed that the choice of facilities influence the level of bureaucracy present in their meetings with emergent adults. Using building stock outside NAV’s offices naturally fosters closer, more informal contact with individuals. Julian, one of the staff members, expressed his relief in escaping the bureaucratic confines of the NAV office, finding liberation in more welcoming facilities.

I’ll die if I have to go back to the NAV office. I hope to avoid that because it’s something else to enter a house like this. Here, I can put away all the bureaucratic stuff and stop thinking about laws and rules. It’s completely uninteresting. It’s been so liberating, and I’ve learned at least as much as the participants have.

Meanwhile, participants highlighted the need for versatile facilities that cater to various needs, from accommodating large groups to providing solitude. Kaira emphasized the importance of a flexible environment that respects individual preferences and tasks:

The facilities have to be designed so that it’s possible to be together as a group or just be alone. Because people are different, some like to be with others all the time, while others like to be by themselves. It also depends on your tasks. For example, to write lyrics, most people can’t sit at a big table with others. That’s not possible.

In one of the programs, due to an infestation, participants transitioned from a spacious, multi-zoned house with its own stage to a newer facility. That move, albeit providing access to a larger stage, limited their flexibility because stage use required planning:

The old facility, plagued by rot, mold, and mice, made us sick. We experienced allergies, heavy coughing, and fatigue. But we had the stage to ourselves, which let us work effectively. We could split up, sing, and work with music at our convenience without needing to book the stage. Music bins outside the venue were a great addition. People who preferred to sing without an audience could use them. We thought moving to a newer facility would be better for our health, but it was smaller and required us to book the stage in advance. I missed the old building where we played more music. (Kaira)

Recognizing and valuing the role of the physical setting, participants favored thoughtfully designed and adaptable facilities. Tailored facilities are crucial for fostering informal interactions and reducing bureaucracy, as noted by participants and staff. Versatile facilities that cater to both group activities and individual needs enhance the dynamics of giving and receiving help.

## 4 Discussion

The study presented in this article aims to determine the defining elements of helpful help within art-based vocational rehabilitation programs in labor and welfare services as experienced and articulated by NEET individuals and staff. The analysis identified three key constituents of helpful help (1): balancing demands and support for personal growth (2), a healing environment with supportive staff, and (4) tailored facilities. Although these constituents provide distinct perspectives, they are intricately interwoven and interact within the exploration of the lifeworld, offering a comprehensive understanding of the phenomenon of helpful help. Together, these constituents indicate that helpful help emerges dynamically, intricately woven through the emergent adult’s progression at their own pace. This process is further influenced by the staff’s knowledge and attunement to their role, as well as the environment and facilities that embrace NEET individuals’ “being-in-the-world,” to borrow Heidegger’s term. Thus, help is not merely a static offering but the result of a dynamic interplay. In this context, the discussion delves into the significance of supportive and non-judgmental relationships with staff, the impact of time and the influence of physical environment and nurturing working atmosphere in youth work. These elements are analyzed in relation to existing research.

### 4.1 The significance of supportive and non-judgmental relationships with staff

In the context of ABVR, relationships are fundamental to vocational support. This study shows that helpful help is rooted in the relational depth between staff and NEET individuals. Cultivating these relationships requires diligence and prioritized commitment to the individuals within the program, which is vital for strengthening these bonds. In turn, such relationships serve as the cornerstone for vocational rehabilitation, aligning with Katznelson’s (30) findings on the importance of robust relationship for young people’s motivation.

In line with Fyhn et al. (6), the findings of this study show that NEET individuals value the quality of their relationships with staff over professional expertise. The quality of these relationships, enhanced by the staff’s genuine engagement with the individuals’ goals, is crucial in establishing significant bonds and was observed to increase when staff showed genuine interest and hope. Staff members’ deep understanding and genuine caring about emergent adults’ needs and interest is not just beneficial but essential, as is nurturing hope in them, for many have lost confidence in their ability to thrive at work and educational settings. The staff’s supportive role thus transcends the mere meeting of needs; more than that, they are key in instilling individuals’ belief in their own potential.

Conversely, relationships with staff who exhibit stereotypical attitudes and disrespectful behaviors, such as talking behind individuals’ backs and feigning trustworthiness only to betray them later, fostered insecurity. Previous research has shown that staff sometimes hold negative attitudes toward the groups that they are meant to assist, impacting their service delivery (41–43). Ljungberg et al.’s (44) literature review, demonstrates that staff members who act according to a predefined and limited view of individuals and their situations, instead of meeting them as unique human beings, are perceived as unhelpful professionals.

Consistent with Frøyland (43), our study underscores that helpers holding prejudiced opinions about young welfare recipients are counterproductive. Encountering negative attitudes from staff can undermine vocational support programs, highlighting the necessity of selecting staff who are supportive, non-judgmental, reassuring, warm, and caring. These staff members must respect emergent adults, endure rejection, and understand that relapses or fluctuating progress may occur during vocational rehabilitation. Our study shows that when staff exhibit these qualities, they foster trust, making emergent adults feel secure enough to engage in transformational processes. Consequently, the staff’s demeanor significantly influences the individuals’ lived experiences, shaping their self-perception and willingness to participate in rehabilitation. Natland et al.’s (20) study observed that a coaching approach emphasizing strengths rather than deficiencies inspired and motivated emergent adults with follow-up needs in NAV. This was also evident in our study, where staff act as young adults’ “fellow travelers,” offering consistent support and companionship, fostering a unified, mutually enriching journey.

### 4.2 The impact of time

Participants in this study point out time as a critical component of helpful help, one that necessitates personalization. For emergent adults with NEET status to perceive help as being beneficial, the time allocated must permit change and foster readiness to engage in the process. Conversely, when time frames are perceived as constricting, participants in this study see no value in making an effort and avoid further unsuccessful undertakings. This reluctance stems from a desire to avoid scenarios with a high likelihood of failure, which would reinforce negative experiences. The necessity of time thus seems to be thoroughly understood by the emergent adults, who know that a shortage of it induces stress and diminishes their willingness to fully commit, thereby reducing the prospects of successful outcomes. This understanding aligns with Frøyland’s (46) emphasis on the importance of time to extend support and ensure NEET individuals have the latitude to achieve their goals. Support should balance concise action with sufficient time to match their pace, avoiding forced solutions that may not be desired or suitable (20).

In 2023, the Norwegian authorities introduced the “Youth Guarantee”, granting emerging adults access to early intervention and close follow-up by NAV until they are 30 years old (47). This guarantee aims to intensify support for emergent adults by allocating additional time to mentoring NEET individuals, thereby enhancing their employment prospects. Rejecting a one-size-fits-all solution, this approach tailors services to match individuals’ personalities and paces, acknowledging diverse needs that require varying degrees of encouragement and time. Traditionally, external providers have delivered much of the long-term follow-up for the NEET individuals in Norway, thereby limiting NAV staff’s experience with offering close, sustained support (46). Frøyland et al. (45) emphasize that tailored follow-up is essential for promoting inclusion in education, employment, and community life, requiring staff to maintain positivity and resilience over extended periods (46). Tailoring policies towards NEETs is challenging (48). An Irish study by Redmond and McFadden (48), notes that the heterogeneity within the NEET population requires distinct policy approaches. Specifically, they underscore that young people with disabilities need a different policy approach compared to other NEET individuals, emphasizing the necessity of individualized support strategies.

Deegan (49) highlights the importance of flexible follow-up services that can adapt to the fluctuating, often nonlinear nature of recovery processes, noting that services often lack such flexibility. This raises questions about NAV’s approach, even with the “youth guarantee”. Does it permit NEET individuals the time needed to recover without rigid time frames? Given Sadeghi et al.’s (12) findings along with NAV’s lack of flexibility and adjustments to individual needs, prioritizing time on the agenda seems necessary. Additionally, it is worth considering whether intensified follow-up from labor and welfare services might further reduce NEET individuals’ employment prospects, given research indicating that potential employers view job seekers with training backgrounds as relatively desirable (16).

### 4.3 The influence of physical environment and nurturing working atmosphere

The study’s findings indicate that both tailored facilities and a healing environment are core elements of helpful help. NEET individuals and staff appreciated facilities separated from the NAV office and situated in community buildings. This setup cultivated an authentic, informal, nonhierarchical environment, fostering a robust sense of community among staff and NEET individuals. This dynamic corroborates Wrede-Jäntti and Wester’s (50) findings on the significant impact of the physical environment on follow-up processes. A relaxed, everyday, non-hierarchical physical environment influences staff’s approach to engaging with emergent adults, shifting their primary task to embodying caring, reassuring adults genuinely invested in the well-being of those they serve, rather than adhering to a fact-focused or bureaucratic professional demeanor. This environment enhances the individuals’ experiences of being acknowledged, signaling that they are viewed as people with potential, not resource-deficient clients. An environment Lee et al. (24) titles empowering for the recipients.

In this study, a nurturing workspace, characterized by a belief in the individuals’ potential, was paramount. This atmosphere became fertile ground where NEET individuals, previously isolated from communal bonds, could cultivate a belief in the possibility of change. They were welcomed into the fold, that is, a community mirroring the collaborative spirit typically reserved for traditional work settings. In this shared space, the seeds of transformation were sown, allowing them to grow and flourish within the collective embrace. Research by Rogstad (51) supports the idea that flexible learning environments can turn young people from “zero to hero” by fostering a sense of community and connection. Contrary to Wrede et al. (51), who advise new, stylish spaces, the findings suggest that welcoming, health-conscious spaces are more important than sleek, modern ones. A well-designed environment should be multifunctional, catering to the various requirements of NEET individuals. It should foster engagement with the community while offering secluded areas for private work. Such a space can provide flexibility for emergent adults to step back from group activities when needed, whether due to fatigue, a preference for smaller groups, or the need for quiet concentration to make progress in their endeavors.

## 5 Implications

The implications of the study’s findings are manifold and invite both practical engagement and scholarly contemplation. Foremost, labor and welfare services are called to honor the lived experiences of NEET individuals and advocate a highly customized, person-centered approach in follow-up. Furthermore, staff should actively cultivate and sustain empathy, nurturing skills, and authentic engagement techniques to foster supportive, judgment-free relationships with emergent adults with social and/or mental health issues. The design of vocational rehabilitation support has to create spaces that enable emergent adults to engage in both social interaction and personal solitude. Future research should investigate the long-term impact of such approaches on NEET individuals’ life stories by delving into how helpful help has shaped their life trajectories over time.

## 6 Strengths and limitations

Due to the limited literature on what constitutes helpful help for emergent adults with mental health and social issues out of education and work, referred to as NEETs within vocational support, this study offers a valuable contribution. The participants have all engaged in art-based vocational programs and other labor and welfare initiatives. Consequently, they sometimes contrast their experiences to highlight what is considered beneficial in art-based vocational rehabilitation programs, which might confuse the reader. Additionally, perspectives from both former and current ABVR participants can be seen as both a limitation and a strength. Former participants provide retrospective insights, which may lack nuance, while current participants might be too close to NAV staff, potentially presenting an idealized view. Nevertheless, the diversity in age, gender, experience, and issues can provide a more nuanced and comprehensive understanding of the phenomenon of helpful help. As a phenomenological study, the sample size is intentionally small to develop in-depth knowledge of the defining elements of helpful help. The strength of this study lies in the rich, detailed descriptions of the phenomenon, revealed through the lived experience of NEET individuals and staff in labor and welfare services. In line with phenomenological approaches, the aim is to broaden understanding rather than explain or generalize findings. However, future research should strive for larger sample sizes and comparative studies to enhance robustness and generalizability, and further validate and expand upon these findings. We suggest incorporating standardized metrics and longitudinal studies to provide a more comprehensive understanding of defining elements, processes, and outcomes of art-based vocational rehabilitation programs.

## 7 Concluding remarks

This study contributes to the understanding of “helpful help” as a lived experience that varies with each unique individual. It reveals that helpful help is a phenomenon that unfolds in the interplay between an individual’s personal journey, the temporal depth of support, and the relational familiarity with the people who aid them. The study highlights that helpful help is embodied in a work environment that breathes optimism and nurturance and where staff engage authentically in fostering relationships that are supportive and free from judgment.

Moreover, the study underscores the importance of embracing a strength-based focus, ensuring that the utilized spaces are welcoming and have the potential to enhance health and well-being. In its most profound sense, helpful help is the crafting of support systems attuned to the singular rhythms and narratives of each person’s life story. For emergent adults with NEET status facing persistent social and mental health issues, it becomes clear that they often have limited self-belief and lack experience of interacting socially with peers as well as other adults. This has left them unready for a sudden transition back into school or employment. For them, gaining experience among peers is crucial for developing self-esteem and skills. The findings call for a significant shift in labour and welfare services, emphasizing the need to go beyond merely offering jobs. The focus should be on providing ongoing personalized support, dedicating sufficient time to keep individuals engaged, and ensuring that the help provided is customized and thorough. This approach may require staff to maintain support for an extended period, but this investment has the potential to make the help helpful for NEET individuals experiencing mental health and social issues.

## Data Availability

The datasets presented in this article are not readily available because we cannot share transcripts from this study. Requests to access the datasets should be directed to kristin.b.orjasater@nord.no.
